# Trace Acetylene Gas Detection Based on a Miniaturized Y-Sphere Coupled Photoacoustic Sensor

**DOI:** 10.3390/s24227274

**Published:** 2024-11-14

**Authors:** Xiaohong Chen, Sen Wang, Dongming Li, Zhao Shi, Qiang Liang

**Affiliations:** Department of College of Automation, Shenyang Institute of Engineering, Shenyang 110136, China; cxh830915@126.com (X.C.); ldm_sut_0312@163.com (D.L.); chessplayer39@163.com (Z.S.); tlliangqiang@163.com (Q.L.)

**Keywords:** Y-sphere coupled photoacoustic sensor, photoacoustic spectroscopy, C_2_H_2_ gas detection

## Abstract

In this work, a miniaturized Y-sphere coupled photoacoustic (YSCPA) sensor is proposed for trace C_2_H_2_ gas detection. The cavity volume of the designed YSCPA sensor is about 0.7 mL. The finite element method (FEM) has been performed to analyze the comparative performance of the YSCPA sensor and T-type PA sensor, indicating that the first-order resonance frequency (FORF) of the newly proposed YSCPA sensor has been reduced by half while the PA signal has been improved by a factor of 3 compared to the T-type PA sensor. C_2_H_2_ is employed as a target gas to test the performance of the YSCPA sensor. The experimental test results show that the response time of the gas is 26 s. The minimum detection limit (MDL) reaches 189 ppb at a lock-in integration time of 1 s. By extending the lock-in integration time to 100 s, the MDL of the designed PA sensor is reduced to 18.1 ppb. The designed YSCPA sensor has the advantages of small size, low gas consumption, simple structure, and high sensitivity, which is expected to be an effective solution for rapid and real-time monitoring of dissolved C_2_H_2_ gas in transformer oil.

## 1. Introduction

Dissolved gas analysis (DGA) in oil has been widely recognized as a reliable method for detecting initial transformer faults [1,2]. The concentration of dissolved acetylene (C_2_H_2_) gas in the transformer oil will be abnormal at the early stage of partial discharge faults in the transformer. Therefore, real-time detection of C_2_H_2_ gas concentration can predict possible faults in transformers. Traditional methods for detecting characteristic gas concentrations in transformers include gas chromatography detection [3,4] and the gas sensor method [5]. The gas chromatography detection method has the disadvantages of a long response time, a complicated operation process, bulky volume, and the need for the column to be replaced periodically. The disadvantages of the gas sensor method for transformer fault gas monitoring are its poor selectivity, low sensitivity, and gas cross-sensitivity. Therefore, neither of the above gas detection methods are suitable for the real-time monitoring of transformer characteristic gas concentrations. However, photoacoustic (PA) spectroscopy (PAS) has the advantages of small size, simple system, high sensitivity, short response time and strong selectivity, which makes it an effective tool for real-time detection of dissolved gases in transformer oil [6,7,8,9,10,11]. Photoacoustic spectroscopy is based on the principle of the PA effect for trace gas detection, which can be summarized as the absorption of light by a gas causing a periodic heat release, which ultimately produces a photoacoustic signal that is detected by a microphone. To date, various concepts have been proposed for gas sensing, such as quartz-enhanced PAS (QEPAS) [12,13,14,15] and cantilever-enhanced PAS (CEPAS) [16,17,18,19]. However, these enhanced PAS technologies are susceptible to temperature and humidity effects that can lead to measurement errors in gas concentrations.

There is a need to develop a photoacoustic cell (PAC) resonance-enhanced PAS for real-time gas detection. Wang et al. reported an H-PAC resonance-enhanced PAS for trace C_2_H_2_ detection [20]. In order to further enhance the sensitivity of PAS, Zhang et al. proposed a novel ellipsoid PAC resonance-enhanced PAS, which can enhance the photoacoustic signal amplitude compared with the H-PAC photoacoustic cell resonance-enhanced PAS [21]. In order to further reduce the response time of the gas, Gong et al. proposed a T-PAC resonance-enhanced PAS for trace C_2_H_2_ detection [22]. Through simulation analysis and experimental verification, it can be seen that compared with H-type PAC resonance-enhanced PAS, T-PAC resonance-enhanced PAS had shorter response time and higher photoacoustic signal. However, all of the above resonance-enhanced PAS methods operate at less than 2000 Hz, so environmental noise has a significant impact on them. In 2024, Zhu et al. proposed a multi-mechanism enhancement based on the acoustic enhancement of the spherical PAC and mechanical enhancement of the cantilever [23]. The first-order resonance frequency (FORF) of the reported spherical photoacoustic cell was close to 6000 Hz. Therefore, it was affected little by the ambient noise, and the overall signal-to-noise ratio was very good. However, the volume of the spherical PAC was large, and therefore the gas response time was very long. Therefore, it became a challenge to design PA sensors with a short response time, high sensitivity, and high resonance frequency.

In this paper, we propose a miniaturized Y-sphere coupled photoacoustic (YSCPA) sensor for rapid and real-time monitoring of dissolved acetylene gas in transformer oil. The cavity volume of the designed YSCPA sensor is about 0.7 mL. The results of finite element analysis show that the performance of the designed YSCPA sensor is better than that of the conventional T-type PA sensor. Experimental results show that the proposed sensor has an C_2_H_2_ detection limit on the order of ppb and a gas response time of 26 s. The designed YSCPA sensor has the advantages of small size, low gas consumption, simple structure, and high sensitivity, which is expected to be an effective solution for rapid and real-time monitoring of dissolved acetylene gas in transformer oil.

## 2. Sensor Design

**Figure 1:**
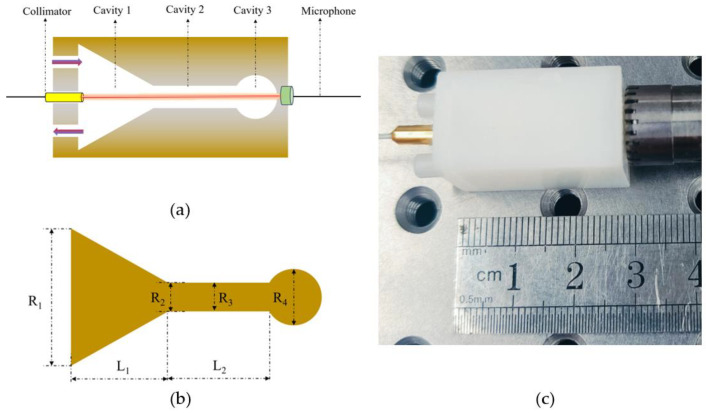
(**a**) The structural schematic of the YSCPA sensor. (**b**) The structural mechanical sketch of the YSCPA sensor. (**c**) The physical drawing of the YSCPA sensor.

Figure 1a–c show the structural schematic, mechanical sketch, and physical drawing of the YSCPA sensor. The sensor includes a collimator, three cavities, and a microphone. The dimensions of cavity 1 are upper radius (R1) = 7.5 mm; bottom radius (R2) = 1.5 mm; and length (L) = 8 mm. The dimensions of cavity 2 are radius (R3) = 1.5 mm and length (L) = 8 mm. The dimensions of cavity 3 are radius (R4) = 3 mm. A hole with a diameter of 1 mm and a length of 0.5 mm is opened at the end of cavity 3 for transmitting the photoacoustic signal to the microphone. The YSPAC is made of photosensitive resin with a density of 1.3g/cm and processed by laser 3D printing technology with a manufacturing accuracy of 0.2 mm. The three-dimensional models of the T-type PA sensor and the YSCPA sensor are established by using the finite element method (FEM). The simulated physical field uses thermo-viscous acoustics for two PA sensors, thereby making the simulation results closer to the experimental test results. In addition, a 1-unit heat source is placed in the buffer chamber portion of two PA sensors to generate the PA signal. The frequency sweep ranges of the T-type PA sensor and the YSCPA sensor are set to 3500–5000 Hz and 8500–10,000 Hz, respectively.

**Figure 2:**
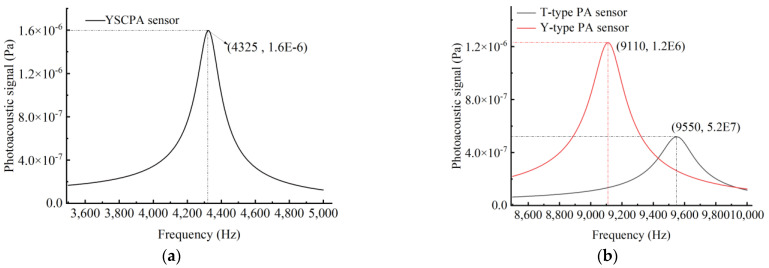
Simulated amplitude–frequency response curves. (**a**) The YSCPA sensor. (**b**) The T-type PA and Y-type PA sensors.

Figure 2 shows the simulated amplitude–frequency response curves of the three PA sensors. Figure 2b shows that the PA signal of the Y-type PA sensor is nearly doubled and the resonance frequency is reduced by 440 Hz compared to the T-type PA sensor. In addition, Figure 2 shows that compared with the T-type PA and Y-type PA sensors, the newly proposed YSCPA sensor has a lower FORF (4325 Hz), as well as a higher PA signal (1.6 × 10^-6^ Pa).

**Figure 3:**
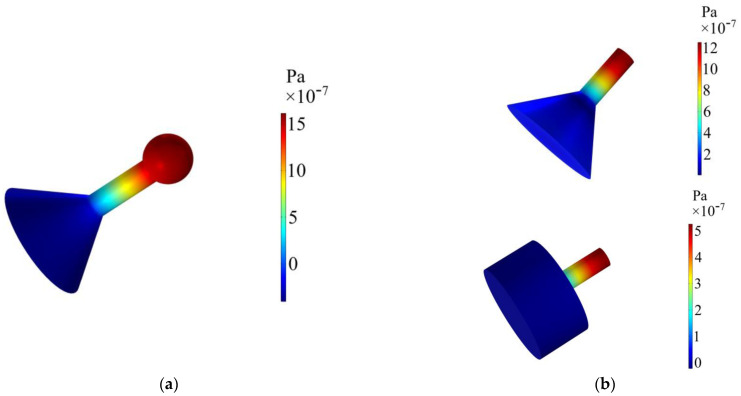
The modal analysis of PA pressure fields. (**a**) The YSCPA sensor. (**b**) The T-type PA and Y-type PA sensors.

Figure 3 depicts the modal analysis of PA pressure fields of the three PA sensors at their FORFs, and it can be observed that the maximum sound pressure of three PA sensors is distributed at the end of the sensor, and thus the condenser microphone is placed here to acquire the maximum sound pressure.

## 3. Experiment Results and Discussion

### 3.1. Instrumentation

**Figure 4:**
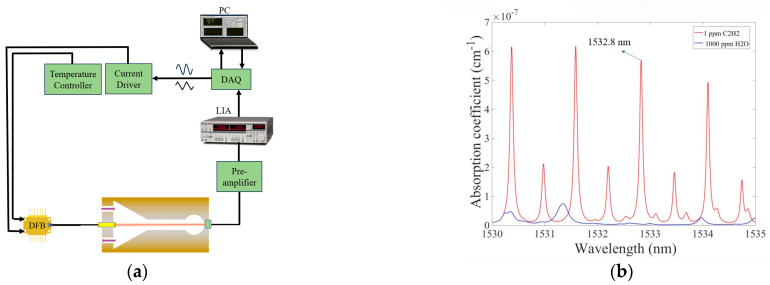
(**a**) Sketch of the experimental test system with the YSCPA sensor. (**b**) Absorption lines of C_2_H_2_ (1 ppm) and water vapor (1000 ppm).

A schematic diagram of the C_2_H_2_ photoacoustic sensor based on Y-sphere coupling is shown in Figure 4. The absorption lines of C_2_H_2_ (1 ppm) and water vapor (1000 ppm) at 1530–1535 nm are shown in Figure 4b. It is observed that the absorption line of acetylene gas at 1532.8 nm is strong and not interfered by water vapour. Therefore, the C_2_H_2_ gas absorption line at 1532.8 nm is selected for testing the sensor performance. The system was equipped with a distributed feedback (DFB) diode laser as an C_2_H_2_ gas excitation source. The laser temperature and drive current are set to 24.9 °C and 62.9 mA, respectively. The erbium-doped fiber amplifier (EDFA) increases the optical power of the DFB laser to 200 mW. The incident beam is processed by the collimator and directly enters the sensor, which generates PA signals after reacting with the C_2_H_2_ gas to be measured, and then is detected by the condenser microphone. Finally, the condenser microphone (B&K 4189, Brüel & Kjær, Nærum, Denmark) collects the electrical signals which are first amplified by a preamplifier and then output to a lock-in amplifier (LIA) (SRS, SR830, Sunnyvale, CA, USA) for processing and transmitted to the computer via a data acquisition card (DAQ).

### 3.2. Experimental Results

**Figure 5:**
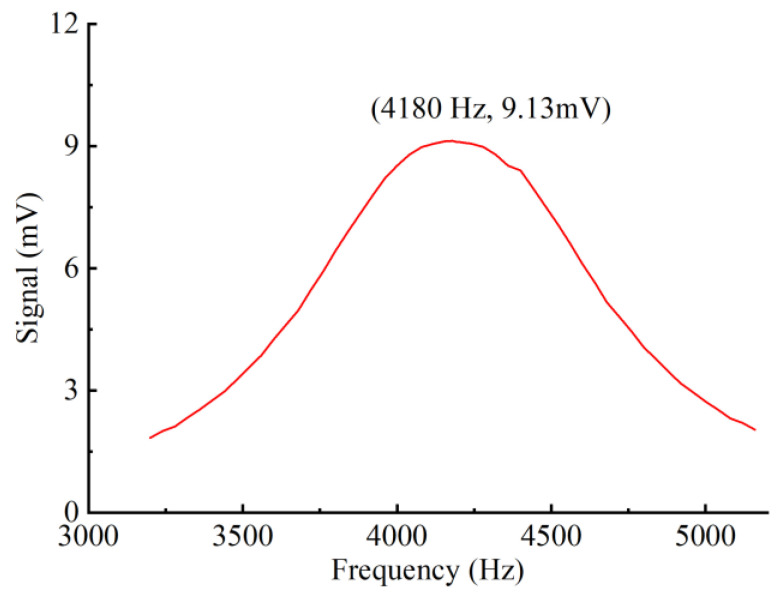
The frequency–response curve of the YSCPA sensor with 2000 ppm C_2_H_2_.

**Figure 6:**
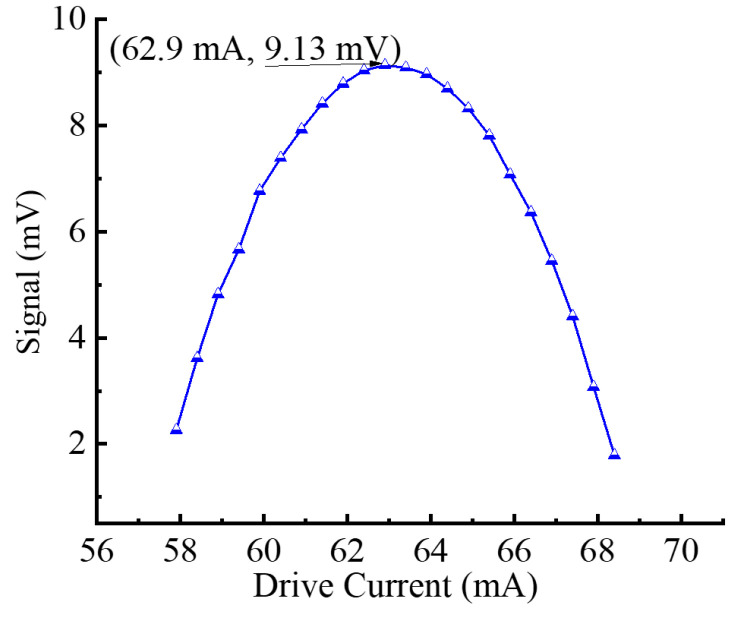
The variation curve of PA signal with modulation current.

The second harmonic (2f) technique is employed to derive the PA signal. For resonant PAS gas sensor systems, the modulation frequency greatly affects the photoacoustic amplitude. Therefore, choosing the right modulation frequency to match the FORF of the YSCPA sensor can obtain an optimal PA signal. The optimal PA signal is obtained by scanning the frequency response curve of the YSCPA sensor with 2000 ppm C_2_H_2_, as shown in Figure 5. The FORF of the YSCPA sensor is observed to be 4180 Hz. The experimental resonant frequency of the designed YSCPA sensor deviates from the simulation results due to the manufacturing error in the coupling part of the 3D printed sensor. In addition, the modulation depth of the sine wave also affects the PA signal. Therefore, it is necessary to choose a suitable modulation depth. Once the operating temperature of the laser does not change, the number of waves emitted by the laser is determined by its injection current. Therefore, the modulation current of the sinusoidal wave is an important factor affecting the PA signal. The variation curve of the PA signal with modulation current is tested, as shown in Figure 6. It can be observed that the PA signal first rises and then decreases with the increase in modulation current, and the PA signal reaches the maximum when the modulation current is 62.9 mA.

**Figure 7:**
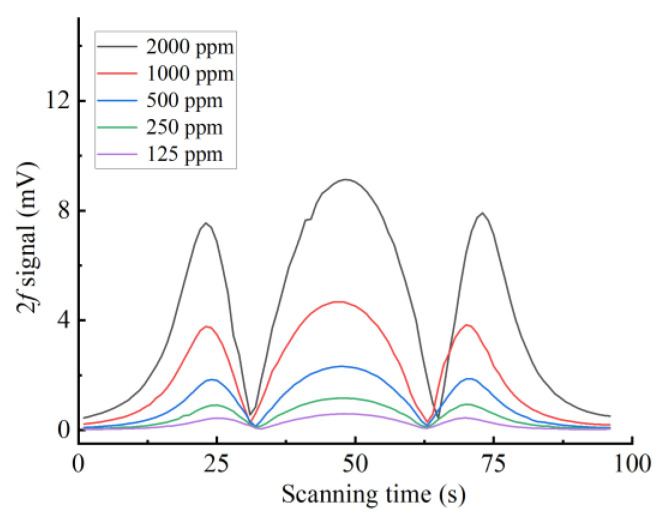
The obtained 2f signal corresponding to different concentrations.

**Figure 8:**
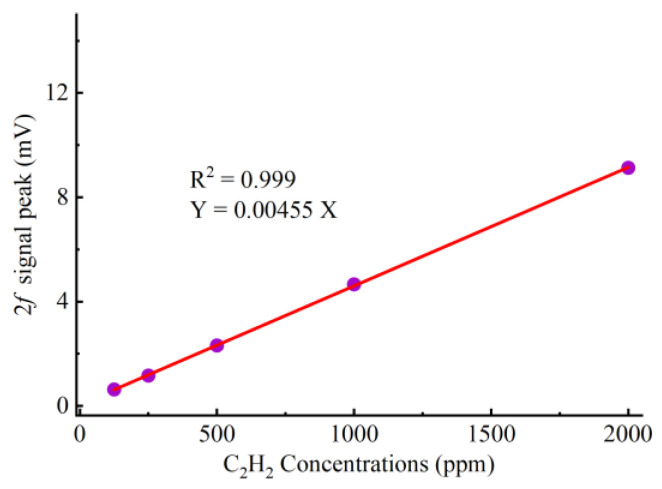
Fitting curves between different C_2_H_2_ concentrations and 2f peak signal.

Two MFCs (CS200, Sevenstar, Beijing, China) are used to control the ratio of 2000 ppm C_2_H_2_/N_2_ gas mixture to 99.99% N_2_ to obtain 125–2000 ppm C_2_H_2_. Different concentrations of C_2_H_2_ (125–2000 ppm) are introduced into the YSCPA sensor to determine the 2f signal of the proposed sensor and the C_2_H_2_ linearity of the sensor. The obtained 2f signals corresponding to different C_2_H_2_ concentrations are shown in Figure 7, and the fitted curves obtained by linear fitting method are shown in Figure 8. It can be observed that the response of the sensor to C_2_H_2_ gas is 4.55 μv/ppm and the R2 reaches 0.999, indicating that the sensor has good linearity for C_2_H_2_.

**Figure 9:**
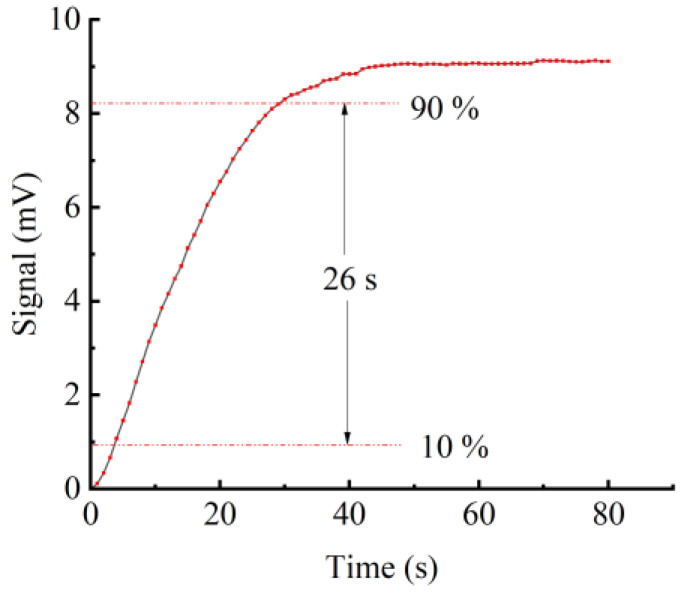
Gas response time of the YSCPA sensor.

The gas response time is an important parameter for PA sensors. Therefore, C_2_H_2_ at a concentration of 2000 ppm is introduced into the PA gas sensor to validate the gas response time of this miniaturized YSCPA sensor. The results of the response time of the test are shown in Figure 9. It can be observed that the photoacoustic signal rises from 10% to 90% in only 26 s, which is sufficient to quickly identify and warn of the dissolved C_2_H_2_ concentration in the transformer oil.

**Figure 10:**
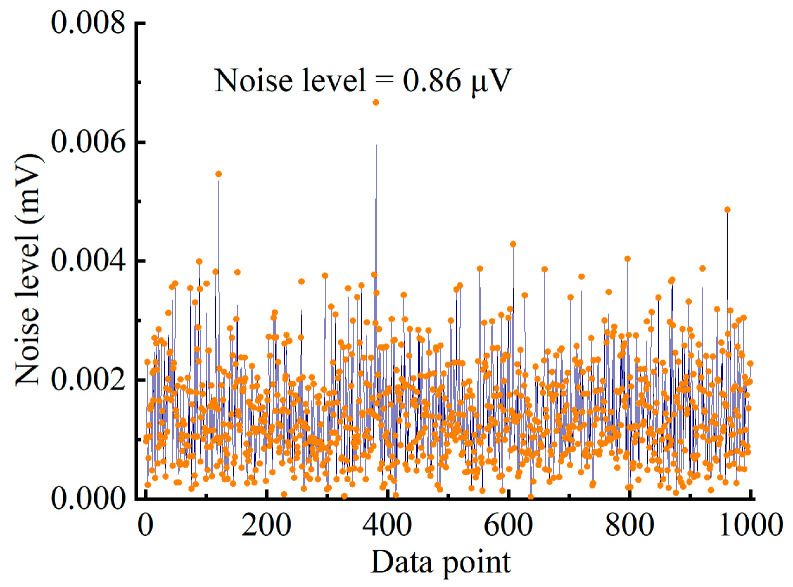
The noise level of the YSCPA sensor-based trace C_2_H_2_ detection system.

The noise level of the system is tested by passing 99.99% nitrogen through the YSCPA sensor for up to one hour until the PA signal no longer degraded. The noise points corresponding to each second of the lock-in amplifier output are continuously recorded, as shown in Figure 10. Therefore, the noise standard deviation of the system is calculated to be 0.86 μv, and the response is 4.55 μv/ppm, yielding an MDL of 189 ppb.

**Figure 11:**
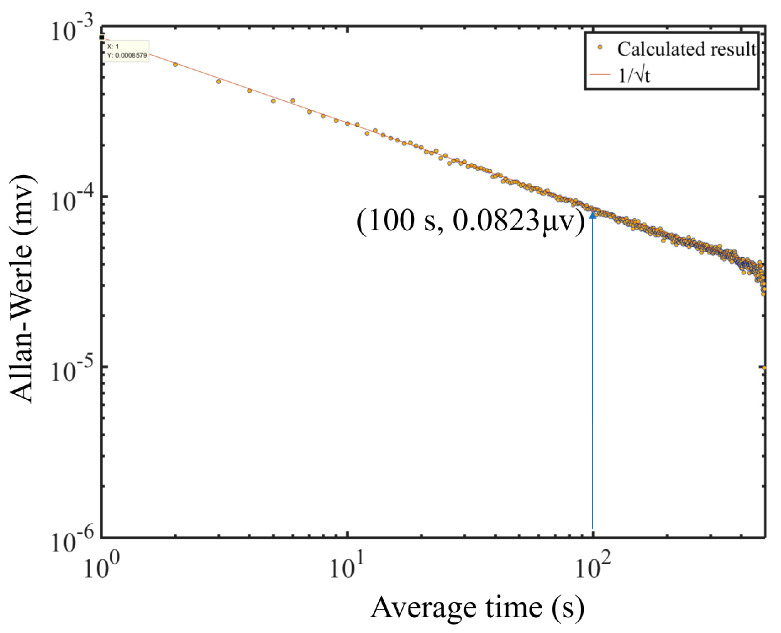
The Allan variance of the YSCPA sensor.

An Allen variance has been employed to assess the long-term stability of the YSCPA sensor system. The results of Allan variance calculation are shown in Figure 11. It can be observed that the Allan variance shows a continuous decreasing trend as the lock-in integration time increases. Therefore, extending the lock-in integration time is one way to improve MDL. The MDL of the designed PA sensor is reduced to 18.1 ppb when the lock-in integration time is 100 s. In addition, the overall trend of Allen variance conforms to the fitted curve with $\sqrt{t}$, which indicates that the system is mainly affected by white noise and therefore has good long-term stability.

## 4. Conclusions

In this work, a miniaturized YSCPA sensor is proposed for trace C_2_H_2_ gas detection. The cavity volume of the designed YSCPA sensor is about 0.7 mL. FEM has been performed to analyze the comparative performance of the YSCPA sensor and T-type PA sensor, showing that the FORF of the newly proposed YSCPA sensor has been reduced by half while the PA signal has been improved by a factor of 3 compared to the T-type PA sensor. The FORF of the newly proposed YSCPA sensor is 4180 Hz, which agrees well with the simulation results. Thanks to the sub-milliliter cavity volume of the designed sensor, the response time of the gas is only 26 s. The MDL reaches 189 ppb at a lock-in integration time of 1 s. The Allen variance results show good stability of the sensing system and provides an idea to reduce the gas detection limit by extending the lock-in integration time. The designed YSCPA sensor has the advantages of small size, low gas consumption, simple structure, and high sensitivity, which is expected to be an effective solution for rapid and real-time monitoring of dissolved C_2_H_2_ gas in transformer oil.

## Data Availability

Data is contained within the article.
